# CRISPR/Cas9 gene editing in induced pluripotent stem cells to investigate the feline hypertrophic cardiomyopathy causing *MYBPC3/*R820W mutation

**DOI:** 10.1371/journal.pone.0311761

**Published:** 2024-10-10

**Authors:** Luke C. Dutton, Jayesh Dudhia, Deborah J. Guest, David J. Connolly

**Affiliations:** Department of Clinical Science and Services, Royal Veterinary College, Hatfield, London, United Kingdom; Okayama University: Okayama Daigaku, JAPAN

## Abstract

Hypertrophic cardiomyopathy (HCM) is the most common heart disease in domestic cats, often leading to congestive heart failure and death, with current treatment strategies unable to reverse or prevent progression of the disease. The underlying pathological processes driving HCM remain unclear, which hinders novel drug discovery. The aim of this study was to generate a cellular model of the feline HCM-causing *MYBPC3* mutation R820W. Using CRISPR/Cas9 gene editing we introduced the R820W mutation into a human induced pluripotent stem cell (iPSC) line. We differentiated both homozygous mutant clones and isogenic control clones to cardiomyocytes (iPSC-CMs). Protein quantification indicated that haploinsufficiency is not the disease mechanism of the mutation. Homozygous mutant iPSC-CMs had a larger cell area than isogenic controls, with the sarcomere structure and incorporation of cMyBP-C appearing similar between mutant and control iPSC-CMs. Contraction kinetic analysis indicated that homozygous iPSC-CMs have impaired relaxation and are hypocontractile compared to isogenic control iPSC-CMs. In summary, we demonstrate successful generation of an iPSC model of a feline *MYBPC3* mutation, with the cellular model recapitulating aspects of HCM including cellular hypertrophy and impaired relaxation kinetics. We anticipate that further study of this model will lead to improved understanding of the disease-causing molecular mechanism, ultimately leading to novel drug discovery.

## Introduction

Hypertrophic cardiomyopathy (HCM) is the most common heart disease affecting domestic cats (*Felis catus*), defined as the presence of left ventricular hypertrophy in the absence of abnormal loading conditions [1]. Feline HCM causes significant morbidity and mortality, affecting around 15% of all cats and with almost 30% of these suffering cardiovascular death [2, 3]. Whilst the phenotypic expression of HCM in cats has been extensively studied, drug discovery is hindered by a lack of understanding of the underlying disease mechanisms and of disease models [4]. A recent study using feline heart protein found that HCM cat hearts had a higher calcium sensitivity, and this calcium sensitivity was not modulated by troponin-I phosphorylation (termed uncoupling) [5]. This finding mirrors those in human studies on HCM [6]. In human medicine, progression in this area has been accelerated using induced pluripotent stem cell derived cardiomyocytes, which can either be derived from diseased patients or gene edited to express an HCM-causing mutation [7–9].

Mutations in the cardiac myosin binding protein C (cMyBP-C) gene (*MYBPC3*) account for 40–50% of causal variants identified in human HCM [10, 11]. Over 90% of these mutations produce a premature termination codon (PTC) resulting in nonsense- mediated decay (NMD) of mRNA transcripts and/or ubiquitin-mediated proteasomal (UPS) degradation of misfolded protein, leading to haploinsufficiency [11–13]. However, some missense *MYBPC3* mutations, including the Ragdoll R820W mutation, do not result in a PTC but instead are likely to alter protein function or structure [14, 15]. While the N terminal and C terminal domains of cMyBP-C have a more well characterised role, much less is known about the function of the central domains (C3-C6), where the R820W and other mutations are located [16, 17]. Computational models suggest that folding of the C6 domain causes the R820 residue to be surface exposed and therefore available for protein interaction. Mutations in this residue may therefore affect protein-protein interactions rather than protein folding [18].

Our aim was to develop a cellular model of the Ragdoll R820W mutation in a human iPSC model in order to create a resource for future research into the mechanistic effects of this mutation. This would provide a valuable insight into feline HCM given the similarities in the disease phenotype between humans and cats, in addition the same mutation is known to cause HCM in a human family which exhibit many of the phenotypic characteristics seen in Ragdoll cats [15]. We therefore used our genetically modified human iPSCs to produce a relevant iPSC-CM model of a well- characterised form of feline HCM and compare effects of the mutation on cell phenotype.

## Materials and methods

### Cell culture

The cell line HPSI0114i-kolf_2 (hereafter named kolf_2) was used for gene editing experiments. We acknowledge Wellcome Trust Sanger Institute as the source of HPSI0114i-kolf_2 human induced pluripotent cell line which was generated under the Human Induced Pluripotent Stem Cell Initiative and acknowledges Life Science Technologies Corporation as the provider of Cytotune. Cells were cultured in StemFlex (Thermo Fisher) media on Geltrex™-coated tissue culture plastic (Thermo Fisher) and incubated in standard cell culture conditions (humidified air, 37°C, 5% CO_2_). Cells were passaged at 70–80% confluence using the EDTA method previously described using a split ratio of 1:10 – 1:12 [19].

### CRISPR/Cas9 gene editing

CRISPR/Cas9 gene editing of iPSCs was performed as previously described [20]. Guide RNA (gRNA) was designed using Benchling software (https://benchling.com). Guide sequences were assessed computationally for on-target efficiency and likely off-target effects using Benchling, CRISPOR (http://crispor.gi.ucsc.edu) and CCTop (https://cctop.cos.uni-heidelberg.de). The guide RNA (gRNA) sequence was 5’-GGCCATCGAGAAGAAGAAGA-3’ and was purchased as combined crRNA/tracrRNA as a synthetic single gRNA (Sigma-Aldrich). The single-stranded oligodeoxynucleotide (ssODN) sequence was designed with homology to 50 nucleotides (nt) upstream and downstream of the C to T change. Additional silent SNPs were also incorporated in the gRNA binding site to help prevent rebinding by the Cas9 endonuclease. The additional SNPs are underlined and change an adenine base to a thymidine base:

5’-ATCAGGTCGAAGTTCAGCCACATCCATCTGTAGCTCTTCTTCTTCTTGCGCT-3’.

The ssODNs were purchased as Ultramers (Integrated DNA Technologies (IDT), Coralville, Iowa, USA). Amino acid sequence homology between humans and cats for this region of MYBPC3 is shown in Table 1 as previously reported [14].

**Table 1:** Amino acid sequence homology between humans and cats for the region of MYBPC3 where the R820W mutation is located. Differences in the sequence are shown in **bold**.

Species	Amino Acid Sequence
*Human*	ILERKKKKSYRWM**R**LNFDLL**R**ELSHEARRMIEG
*Feline (wild type)*	ILERKKKKSFRWM**R**LNFDLL**Q**ELSHEARRMIEG
*Feline (mutant)*	ILERKKKKSFRWM**W**LNFDLL**Q**ELSHEARRMIEG

Kolf_2 cells between passages 10 and 15 that were growing for 3–5 days and < 70% confluent were used for nucleofections. The synthetic single gRNA was re-suspended in 66 μL of IDTE buffer (IDT) to make a 45 μM solution. High-fidelity *Sp*Cas9 (Alt-R S.p. Cas9 nuclease V3, IDT) was diluted to 4 μg/μL in Cas9 storage buffer (10 mM Tris-HCl, pH 7.4, 300 mM NaCl, 0.1 mM EDTA, 1 mM dithiothreitol (DTT)). To form the ribonucleoprotein (RNP) complex, 5 μL of *Sp*Cas9 was combined with 5 μL of sgRNA and incubated at room temperature for 10–20 min. Next 500 pmol of ssODN template was added to the RNP complex.

To obtain the iPSCs as single cells, 20 μL of 100X RevitaCell (Thermo Fisher) was added per well of a 6- well plate for 1 h in standard cell culture conditions. Media was removed and cells were washed once with DPBS and 0.75X TrypLE Express (Thermo Fisher, diluted in DPBS) was added for 3–5 min. An equal volume of StemFlex media supplemented with 1X RevitaCell was then added, and cells pipetted to obtain single cells. Cells were transferred to a centrifuge tube, pelleted (300*g* for 3 min) and re-suspended in StemFlex/RevitaCell for counting. Live cells were counted using trypan blue dye exclusion on a Countess II Automated Cell Counter (Thermo Fisher) according to the manufacturer’s instructions. A total of 1 x 10^6^ cells were transferred to a microcentrifuge tube (Greiner Bio-one), pelleted (300*g* for 3 min) and re-suspended in 100 μL of complete P3 buffer from a P3 Primary Cell 4D-Nucleofector X Kit L (Lonza). The ssODN/RNP complex was then added to the cell suspension and transferred to the nucleofection cuvette. The samples were electroporated using a 4D Nucleofector (Lonza) set to program CA 137. Transfected iPSCs were then divided between 2 wells of a 6-well plate and maintained until 70% confluence prior to subcloning.

### CRISPR/Cas9 MiSeq library preparation

Induced pluripotent stem cells underwent single cell cloning by plating cells at low density (~20 cells/cm^2^) in 100 mm tissue culture dishes (Greiner Bio-one) and then manually picking colonies into 96-well plates in duplicates (one clone split between 2 wells in two different 96-well plates). Cells were grown to 80% confluence, at which point cells from one of the plates were washed once with DPBS and then DirectPCR Lysis Reagent (Viagen) was added and incubated for 30–60 s at room temperature. Lysate was collected into a centrifuge tube, incubated at 60°C for 1 h and then at 95°C for 10 min. Lysate was diluted 1:10 with molecular biology grade water (Sigma-Aldrich). To obtain DNA amplicons suitable for next-generation sequencing, a high-fidelity DNA polymerase mix was used (KAPA HiFi HotStart ReadyMix, Roche). A 10 μL reaction volume consisted of 2 μL of lysate, 5 μL KAPA HiFi HotStart ReadyMix, 0.3 μL of each forward and reverse primer and 7.6 μL of water. The forward primer was 5’-CACAGTACAGTGGGAGCCG-3’ and reverse primer was 5’- TGGATGTGAGTGTCTAGCATGG-3’ (amplicon of 452 bp) with the intended CRISPR cut site situated approximately in the middle. PCR was performed in 96-well PCR plates (Greiner Bio-one) using a thermal cycler (CFX96 Real Time PCR Detection System, Bio-rad) with the following settings: 95°C for 3 min, followed by 35 cycles of 98°C for 20 s, 60°C for 15 s and 72°C for 30 s with a final extension at 72°C for 5 min. Amplification was confirmed using a 2% agarose gel electrophoresis.

Then 0.5 μL of this PCR product was used to prepare a nested amplification using the forward primer:

5’-ACACTCTTTCCCTACACGACGCTCTTCCGATCTGTTCCAGACCAGAGCTGCC-3’

### And reverse primer

5’-TCGGCATTCCTGCTGAACCGCTCTTCCGATCTGATAGGCATGAAGGGCTGGG-3’

The proximal parts of the MiSeq Illumina adapter are underlined. PCR conditions were the same. Each sample was then diluted 1:100 in nuclease-free water and 0.5 μL used in the third PCR to generate barcoded amplicons. Illumina i5 and i7 barcoded primers were used for this third PCR. Each 96 well plate had a unique i5 primer barcode and each well of a 96 well plate had a unique i7 primer barcode. The annealing temperature for this PCR was 70°C (other parameters remained the same). After each PCR, 3 μL of product was run on a 96-well 2% agarose gel to confirm successful amplification. After the final PCR, 2 μL of each of the barcoded samples was pooled and purified using AMPure beads according to the manufactures’ instruction (Beckman Coulter). This pooled product was separated using 2% agarose gel electrophoresis and the band corresponding to the predicted amplicon size (367 bp) extracted and purified by using the GenEluteTM Gel Extraction Kit (Sigma-Aldrich) according to the manufacturer’s instructions. The library was then submitted for 150 nt paired-end sequencing at Edinburgh Genomics (Edinburgh University, UK) with a minimum read depth of 1000 reads per sample. The command line utility CRISPResso2 (Pinello laboratory, Massachusetts General Hospital, Charlestown, MA) was used to analyse FASTQ files, with homozygous genotypes determined if the genotype represented >99% of the total reads for that cell clone.

Cell clones that were homozygous (herein termed mutant cells, n = 3) or clones that only contained the blocking mutation (herein termed isogenic controls, n = 3) based on next-generation sequencing were then expanded and re-sequencing of these clones was performed using Sanger sequencing (Eurofins Genomics) to confirm the genotype. Sequences were aligned and analysed using CLC Genomics Workbench version 12 (Qiagen). To form embryos bodies for trilineage differentiation, cells were detached using Dispase (1U mL-1; Thermo Fisher) for 3–5 min, until the edges of the colonies started to lift. Colonies were washed off the plate using DPBS and allowed to sediment by gravity for 5 min. Next, colonies were re-suspended in embryoid body (EB) media, which consisted of KSR based human iPSC media without bFGF. These were then added to CELLSTAR cell-repellent 6-well plates (Greiner Bio-One). After 4–5 days, embryoid bodies were collected and re-distributed onto 0.2% gelatin coated 48-well plates and allowed to differentiate for 3 weeks before antibody staining. Each clone generated was assessed for expression of the pluripotent markers *NANOG*, *Sox2*, SSEA4 and Tra-1-60 as described under immunocytochemistry.

### Cardiac directed differentiation

Previously published protocols [21, 22], optimised for the kolf_2 cell line, were used to differentiate mutant iPSCs (n = 3) and isogenic control iPSCs (n = 3) to cardiomyocytes. Cells were seeded 4 days prior to mesoderm induction in 6-well tissue culture plates (Greiner bio-one) at a density of 1.6 x 10^4^ cell cm^-2^. On day -1, a Geltrex matrix overlay was added to the cells. On day 0, media was changed to cardiac induction media consisting of RPMI 1640 (Thermo Fisher), B27 without insulin (Thermo Fisher), 12 μM CHIR99021 (Sigma-Aldrich) and 10 ng μL^-1^ Activin A (Sigma-Aldrich). Media was changed 24 h later to RPMI with B27 without insulin. On day 3, media was changed to RMPI, B27 without insulin and 2 μM IWP 2 (Sigma-Aldrich). On day 5, media was changed to RPMI with B27 (with insulin), and media changed every 2–3 days. Beating cells were noted from day 8 onwards. Metabolic selection (MS) of iPSC-CMs was carried out as previously described [23]. On day 10 of differentiation, media was changed to MS media consisting of RPMI 1640 without glucose (Thermo Fisher), recombinant human albumin (500 μg mL^-1^; Sigma-Aldrich), L-ascorbic acid 2-phosphate (213 μg mL^-1^; Sigma-Aldrich) and sodium DL-lactate (5 mM; Sigma Aldrich). This was changed every other day for 5 days. Media was then changed to cardiac maintenance media (CMM) consisting of RPMI 1640 with B27 supplement.

### Passaging and use of iPSC-CM in assays

Three days after the cessation of MS, purified cardiomyocytes were dissociated using cell dissociation solution (CDS), which consisted of 40% v/v RPMI 1640, 20% v/v 0.25% trypsin-EDTA (Thermo Fisher) and 40% v/v Cell Dissociation Buffer (Thermo Fisher). First iPSC-CMs were washed twice with DPBS, CDS added, and cells incubated at 37°C for 5 min. Cells were then disrupted by pipetting using a P-1000 tip and incubated for a further 5 min. Cells were collected, added to re-plating media consisting of CMM with 10% v/v fetal bovine serum (Thermo Fisher) and pelleted (100 *g* for 15 min). Cells were re-suspended in re-plating media with 1X RevitaCell added and passed through a 70 μM cell strainer. Cells were then re-plated onto Geltrex coated 6 well plates in CMM, 10% FBS and 1X RevitaCell at a density of 2 x 10^5^ cells cm^-2^. After 48 h media was changed to CMM and cardiomyocytes maintained with media changes every 2–3 days. Cardiomyocytes were used in assays at day 35.

### Quantitative polymerase chain reaction

RNA was extracted from cells using the GenElute Mammalian Total RNA Miniprep Kit (Sigma-Aldrich) according to the manufacturer’s instructions. To remove genomic DNA, RNA was subjected to DNase treatment using the TURBO DNA-*free* Kit (Thermo Fisher) according to the manufacturer’s instructions. RNA was quantified using a spectrophotometer (DS-11 Series, DeNovix Inc.) and a 260/280 ratio of ≈ 2.0 taken to indicate good quality RNA. Then 1 μg of RNA was used for complementary DNA (cDNA) synthesis using the SensiFAST cDNA Synthesis Kit (Bioline) according to the manufacturer’s instructions with a total reaction volume of 20 μL.

Quantitative PCR (qPCR) was performed using 2 μL of cDNA template (equivalent to 40 ng) using the SensiMix SYBR green No-ROX kit (Bioline) and run on a CFX96 Real Time PCR Detection System (Bio-rad) with the following conditions: 95°C for 10 min, then 44 cycles of 95°C for 15 s, 60°C for 15s, 72°C for 15s. A final ramp of 60–90°C at 0.5°C increments was used for melt curve analysis. All samples were run in technical duplicates. Standard curve and melt curve analysis was determined for each primer pair to ensure efficiency and specificity of the reaction. Amplicon size was verified using a 2% agarose gel electrophoresis. To obtain relative abundance of transcripts the double delta cycle threshold (CT) method was used using Alu repeats as the housekeeping gene. This housekeeping gene was selected as it appeared the most stable across the analysed cell populations from 6 original candidate genes (*Alu* repeats, *RPLPO*, *PPIA*, *RPL13A*, *YWHAZ* and GAPDH). The primer sequences are given in Table 2.

**Table 2:** Primer sequences used for qPCR.

Gene	Sequence (5’-3’)	Product size (bp)
*Alu*	F: CATGGTGAAACCCCGTCTCTA	91
	R: GCCTCAGCCTCCCGAGTAG	
*TNNT2*	F: GCGGGTCTTGGAGACTTTCT	93
	R: TTCGACCTGCAGGAGAAGTT	
*ACTC1*	F: TCCCATCGAGCATGGTATCAT	238
	R: GGTACGGCCAGAAGCATACA	
*hNanog*	F: ATGCCTCACACGGAGACTGT	103
	R: AAGTGGGTTGTTTGCCTTTG	
*MYBPC3*	F: CAAGGTCTATCTGTTCGAGCTG	228
	R: AGAATCCCAGTGTCCTCATGG	
*RPL13A*	F: CCTGGAGGAGAAGAGGAAAGAGA	126
	R: TTGAGGACCTCTGTGTATTTGTCAA	
*YWHAZ*	F: ACTTTTGGTACATTGTGGCTTCAA	94
	R: CCGCCAGGACAAACCAGTAT	
*GAPDH*	F: GAGTCAACGGATTTGGTCGT	238
	R: TTGATTTTGGAGGGATCTCG	
*PPIA*	F: TCCTGGCATCTTGTCCAT	179
	R: TGCTGGTCTTGCCATTCCT	
*RPLPO*	F: AATCTCCAGGGGCACCATT	74
	R: CGCTGGCTCCCACTTTGT	

### Immunofluorescence

Cardiomyocytes for immunofluorescence were detached from culture vessels as previously described and seeded onto Geltrex-coated Lab-Tek II four-chamber slides (Thermo Fisher) at a density of 2 x 10^5^ cells cm^-2^ in CMM with 10% FBS and 1X RevitaCell. Cells were cultured for 3 days and media (CMM) was changed every day. The same process was followed for induced pluripotent stem cells except using EDTA passaging and seeding onto Geltrex-coated chamber slides in StemFlex media. Immunostaining procedures were performed at room temperature following removal of culture media. Cells were washed once with DPBS and fixed in 4% paraformaldehyde (Sigma-Aldrich) for 15 min. Fixative was then removed and cells permeabilised using 0.4% Triton X-100 (Sigma-Aldrich) for 15 min. Cells were washed with 0.05% Tween 20/DPBS (Sigma Aldrich) and 10% goat serum (Abcam) added to block non-specific binding of antibodies for 1 h. Cells were washed again and incubated with the primary antibody for 1.5 h. Following three washes cells were incubated with the secondary antibody for 1 h. The antibodies and dilutions used are shown in Table 3. Cells were then washed again three times and nuclei counterstained using NucBlue ReadyProbe according to the manufacturer’s instructions (Thermo Fisher). Wells were removed according to the manufacturer’s instructions and coverslips (Thermo Fisher) applied using VECTASHIELD mounting medium for fluorescence (Vector Laboratories). Images were captured using a Nikon Ti2-E inverted fluorescence microscope.

**Table 3:** Antibodies used in this study.

Antigen	Primary or secondary	Clonality	Host	Reactivity	Supplier	Conjugate	Dilution
Troponin T	Primary	Monoclonal	Mouse	Human	Abcam; ab8295	None	1:100
cMyBP-C	Primary	Monoclonal	Mouse	Human	Santa Cruz Biotechnology; sc-137180	None	1:100
GATA4	Primary	Polyclonal	Rabbit	Human	Abcam; ab84593	None	1:100
α-sarcomeric actinin	Primary	Polyclonal	Rabbit	Human	Abcam; ab137346	None	1:100
Sox2	Primary	Polyclonal	Rabbit	Human	Abcam: ab97959	None	1:200
Nanog	Primary	Polyclonal	Rabbit	Human	Abcam: ab21624	None	1:50
SSEA4	Primary	Monoclonal	Mouse	Human	Stem Cell Technologies; 60062	None	1:100
Tra-1-60	Primary	Monoclonal	Mouse	Human	Biolegend; 330602	None	1:100
Smooth muscle actin	Primary	Monoclonal	Mouse	Human	Sigma; A2547	None	1:100
βIII-tubulin	Primary	Polyclonal	Rabbit	Human	Abcam; ab18207	None	1:100
Insulin	Primary	Monoclonal	Mouse	Human	Abcam; ab6995	None	1:100
IgG	Secondary	Polyclonal	Goat	Rabbit	Abcam; ab150078	Alexa Fluor 555	1:1000
IgG	Secondary	Polyclonal	Goat	Mouse	Biolegend; 405319	Alexa Fluor 488	1:1000
N/A - isotype control	Primary	Polyclonal	Rabbit	N/A	Abcam; ab37415	None	1:100
N/A - isotype control	Primary	Monoclonal	Mouse	N/A	Abcam; ab170190	None	1:100

### Flow cytometry

Triplicate wells of cells of iPSC-CMs were released from culture plastic using trypsin- EDTA, pelleted (400 *g* for 5 min) and re-suspended 4% paraformaldehyde (PFA; Sigma-Aldrich). These were incubated at room temperature for 10 min, pelleted (400 *g* for 5 min) and re-suspended in 0.4% Triton X-100 (diluted in DPBS; Sigma-Aldrich). The cells were pelleted once more and were re-suspended in FACSFlow (BD Bioscience) containing the primary antibody and incubated at RT for 1 h. Cells were then washed once with DPBS and re-suspended in FACSFlow containing a fluorophore labelled secondary antibody for 1 h at RT and then washed for a final time in DPBS and resuspended in FACSFlow for analysis. Undifferentiated iPSCs and isotope labelled cells served as negative controls to set gating parameters. The primary and secondary antibodies used are listed in Table 2. Samples were acquired in polystyrene fluorescence-activated cell sorting (FACS) tubes on a BD LSRFortessa flow cytometer (BD Bioscience). The instrument was calibrated using BD Cytometer Setup and Tracking Beads (BD Bioscience) before acquiring and analysing each set of samples using CellQuest Pro software (BD Bioscience). Control samples were acquired to set the forward and side scatter parameters to centre the cell population on the scatter plot. Fluorescence intensity was adjusted to set the control samples within 100 – 10^1^ on the log scale axis. Cells were then acquired with an event count set to a total of 1 x 10^4^ events. Data was analysed using FlowJo software (FlowJo, Version 10, LLC).

### Measurement of cell size

Cardiomyocytes stained for α-sarcomeric actinin were analysed for cell area, as previously reported [24, 25]. Image J (National Institute of Health) was used to draw the outline of each cell created by the α-sarcomeric actinin staining (as previously validated [24]) and cell area determined by the program. Approximately 100 cells per iPSC-CM cell line were analysed by analysing all cells present within 8–10 randomly acquired images.

### Enzyme-linked immunosorbent assay

Whole cell protein was extracted from day 35 iPSC-CMs using the M-PERTM Mammalian Protein Extraction Reagent according to the manufacturer’s instructions (Thermo Fisher). Total protein concentration was determined for each sample by using the PierceTM BCA Protein Assay Kit according to the manufacturer’s instructions (Thermo Fisher) measured on an Infinite M Plex microplate reader (Tecan Life Sciences). The quantity of cMyBP-C was determined by using the Human MyBPC3 DuoSet ELISA according to the manufacturer’s instructions (R&D Systems) measured on the microplate reader. For each sample, 20 μg of total protein was added per well of the 96-well ELISA plate (Costar, Corning) and run in technical duplicates. The amount of cMyBP-C in each sample was calculated by MyAssays (https://myassays.com, MyAssays Ltd.) using a four-parameter logistic curve produced by 2-fold dilution standards included with the kit. Results are expressed as pg of cMyBP-C in one μg of total protein extract.

### Contraction kinetics

The iPSC-CMs were cultured for 3 days on glass slides and then videos were captured at 50–60 frames per second (fps) using a x40 objective lens on a Nikon Ti2-E inverted microscope within an incubator chamber maintained at 37°C and images captured within an hour of the cell culture being at 0% CO2 to minimise pH change of the media. All cells within the field of view were analysed (100–200 per cell line). Video files were processed and analysed using the open-access ImageJ plugin software package MUSCLEMOTION according to the developer’s instructions (developed by Sala *et al*. [26, 27]). The authors describe the principle behind the software in their paper as follows: “The principle underlying the algorithm of MUSCLEMOTION is the assessment of contraction using an intuitive approach quantifying absolute changes in pixel intensity between a reference frame and the frame of interest. For every pixel in the frame, each reference pixel is subtracted from the corresponding pixel of interest, and the difference is presented in absolute numbers. Unchanged pixels result in low (black) values, whereas pixels that are highly changed result in high (white) value. Next, the mean pixel intensity of the resulting image is measured. This is a quantitative measure of how much the pixels have moved compared with the reference frame: more white pixels indicate more changing pixels and, thus, more displacement. When a series of images is analyzed relative to the same reference image, the output describes the accumulated displacement over time. However, if a series of images is analyzed with a reference frame that depends on the frame of interest, this results in a measure of the relative displacement per interframe interval, defined as contraction velocity”.

### Statistical analysis

The SPSS software package (IBM, version 23 for Mac) and Prism package (GraphPad Software, version 8 for Mac) were used for statistical analysis. Data was assessed for normality using histogram analysis. Unless otherwise stated, all data is presented as the mean ± SEM of three individual clonal iPSC lines that had the same genotype (either homozygous mutant or isogenic control), with samples run at least in triplicate. Comparisons between two independent samples were performed using Student’s two-tailed T-test and between three or more groups using one-way ANOVA with post-hoc Tukey analysis. A p value of < 0.05 was considered significant.

## Results

### R820W mutation editing into iPSC using CRISPR/Cas9

**Fig 1:**
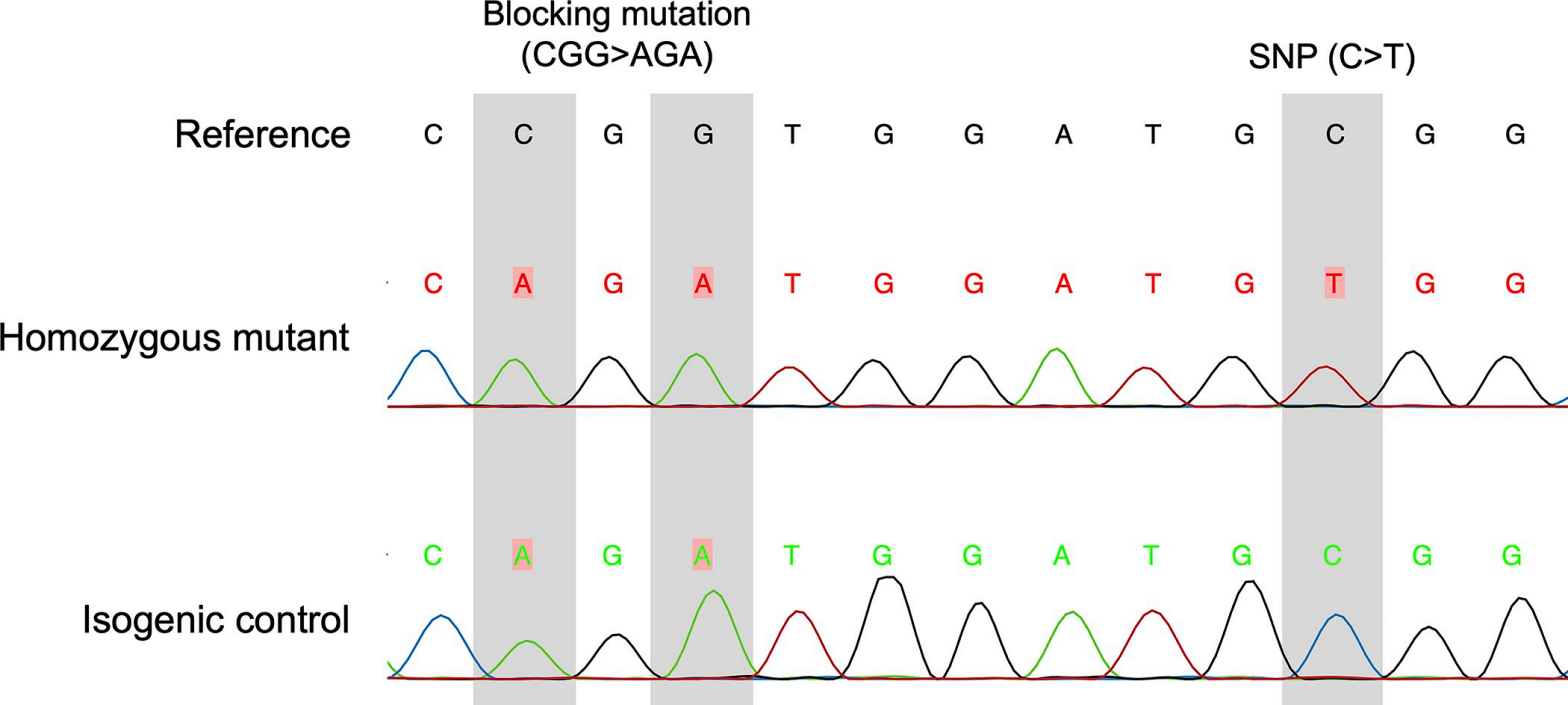
Chromatogram from Sanger sequencing of a representative homozygous mutant clone and an isogenic control cell line. The reference sequence if given at the top (location hg38 47337533 to 47337545). The site of the blocking mutation (to prevent re-cutting of the Cas9 nuclease) and the single nucleotide polymorphism (SNP) that results in the R820W mutation are highlighted. The isogenic control clone has the blocking mutation and is wild-type at the SNP location.

Computational analysis of gRNA sequences indicated that the selected guide had a high specificity with low predicted off-target effects (MIT specificity score of 79 [28] and a CFD specificity score of 79 [29]). The top five (based on CFD score) off-target predicted sites were intergenic (4 sites) and intronic (1 site). Next-generation sequencing analysis revealed that 35 out of 192 selected cell clones were homozygous for the R820W mutation (18.2%). Three clones were selected and repeat sequencing using Sanger sequencing confirmed the homozygous mutation (Fig 1). In addition, clones (n = 3) that only had the blocking mutation inserted (without the R820W mutation) were also selected and re-genotyped confirming their mutation (Fig 1). All clones generated showed the same expression of pluripotent stem cell markers as prior to gene editing and retained the ability to differentiate into cells representing three germ layers (S1 Fig).

### High purity iPSC-CMs generated after metabolic selection

**Fig 2:**
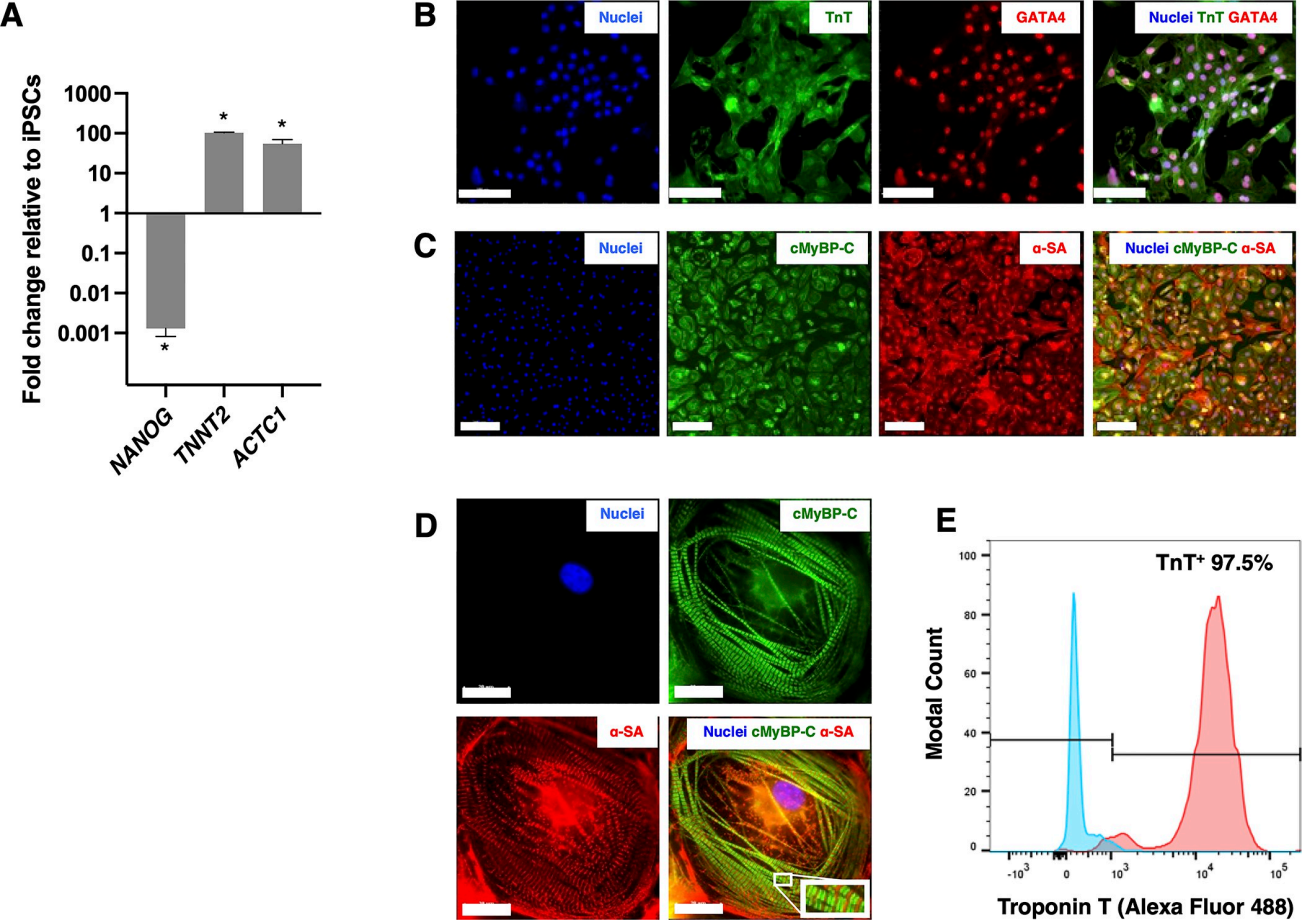
Characterisation of human induced pluripotent stem cell-derived cardiomyocytes (iPSC-CMs) assessed at day 35 of differentiation. RT-qPCR showed that iPSC-CMs significantly down-regulated *NANOG* expression and up-regulated the cardiac specific markers *TNNT2* and *ACTC1* compared to iPSCs (n = 3 iPSC-CMs and 3 iPSC clonal lines from the same donor, p < 0.001, A). The Alu repeat sequences were used as internal control. Immunocytochemistry (ICC) showed that iPSCs expressed troponin T (TnT, green fluorescence, B), GATA4 (red fluorescence, B), cardiac myosin binding protein C (cMyBP-C, green fluorescence, C) and alpha-sarcomeric actinin (α-SA, red fluorescence, C). The iPSC-CMs also had organised sarcomeres, demonstrated by ICC staining of the A-band protein cMyBP-C (green fluorescence, D) and Z-disc protein α-SA (red fluorescence, D). Flow cytometry analysis of iPSC-CMs showed >95% of cells were troponin T positive (n = 3 separate differentiations, red histogram, E) compared to the negative control (undifferentiated iPSCs, blue histogram, E). Scale bars = 100 μm, (B), 200 μm (C) and 20 μm (D). Nuclei are stained with DAPI (blue fluorescence). * = p < 0.001.

Next, iPSCs from each clone (homozygous mutant, n = 3, and isogenic control, n = 3) were differentiated to iPSC-CMs for downstream assays and a subset assessed for cardiac differentiation and phenotype. RT-qPCR analysis showed that iPSC-CMs down-regulated *NANOG* expression compared to iPSCs (p < 0.001), and up-regulated the cardiac-specific markers *TNNT2* (encoding cardiac troponin T2) and *ACTC1* (encoding cardiac α-sarcomeric actin) approximately 100-fold compared to iPSCs (n = 3 sets of differentiation of the iPSCs, p < 0.001, Fig 2A). The iPSC-CM expressed cardiac proteins troponin T, GATA4, cMyBP-C and α-sarcomeric actin (Fig 2B and 2C). These cardiomyocytes displayed organised sarcomeres indicated by arrangement of the z-disc protein α-sarcomeric actinin and A-band protein cMyBP-C (Fig 2D). The iPSC-CMs were obtained with high purity, as >95% of the cell population expressed cardiac troponin T as assessed by flow cytometry (n = 3 separate differentiations, Fig 2E). The remainder of the differentiations were subsequently assessed for cardiac troponin T (cTnT) staining and only included in analysis if cTnT was >95%.

### The expression of cMyBP-C was unaffected in homozygous mutant iPSC-CMs

**Fig 3:**
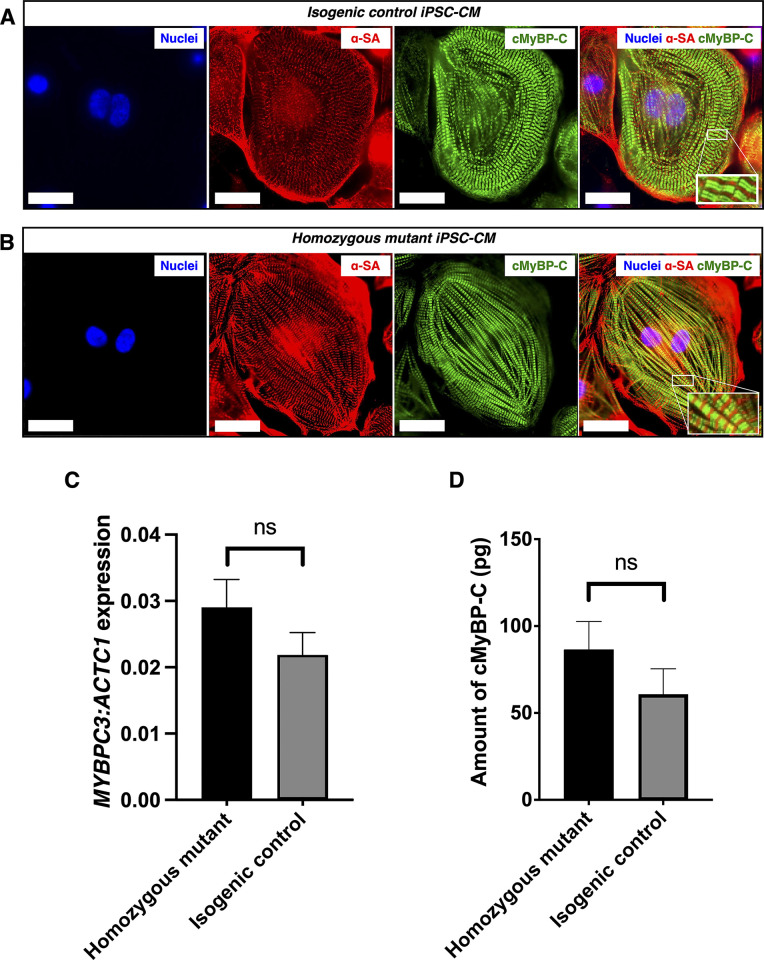
Cardiac myosin binding protein C expression in induced pluripotent stem cell-derived cardiomyocytes (iPSC-CMs) from homozygous mutant and isogenic control human iPSCs. Immunocytochemistry showed that both homozygous mutant and isogenic control iPSC-CMs express and incorporate cardiac myosin binding protein C (cMyBP-C, green fluorescence) into the A-band of the sarcomere between the Z-disc protein alpha-sarcomeric actinin (α-SA, red fluorescence, A and B). Nuclei are stained with DAPI (blue fluorescence). RT-qPCR of *MYPBC3* transcripts relative to *ACTC1* showed no difference in expression levels between homozygous mutant and isogenic control iPSC-CMs (n = 3 homozygous mutant lines and 3 isogenic control lines, p = 0.231, C). The amount of cMyBP-C, measured per μg of total protein extract by ELISA, was also not different between the homozygous mutant and isogenic control iPSC-CMs (n = 3 homozygous mutant lines and 3 isogenic control lines, p = 0.109, D). Scale bars = 20 μM. ns = not significant.

We next sought to assess the degree and organisation of cMyBP-C in the sarcomeres of cardiomyocytes obtained from homozygous mutant and isogenic control iPSCs. Both isogenic control and homozygous mutant cells expressed cMyBP-C protein when analysed using immunocytochemistry (Fig 3A and 3B). The organisation of the sarcomere and incorporation of mutant cMyBP-C appeared the same between homozygous mutant and isogenic control iPSC-CMs. There was no difference in the expression levels of *MYBPC3* relative to *ACTC1* as assessed by qPCR (n = 3 isogenic control lines and n = 3 homozygous lines, p = 0.231, Fig 3C). The amount of cMyBP-C, measured per μg of total protein extract by ELISA, was also not different between the homozygous mutant and isogenic control iPSC-CMs (n = 3 isogenic control lines and n = 3 homozygous lines, p = 0.109, Fig 3D).

### Homozygous mutant iPSC-CMs were larger than isogenic control cells

**Fig 4:**
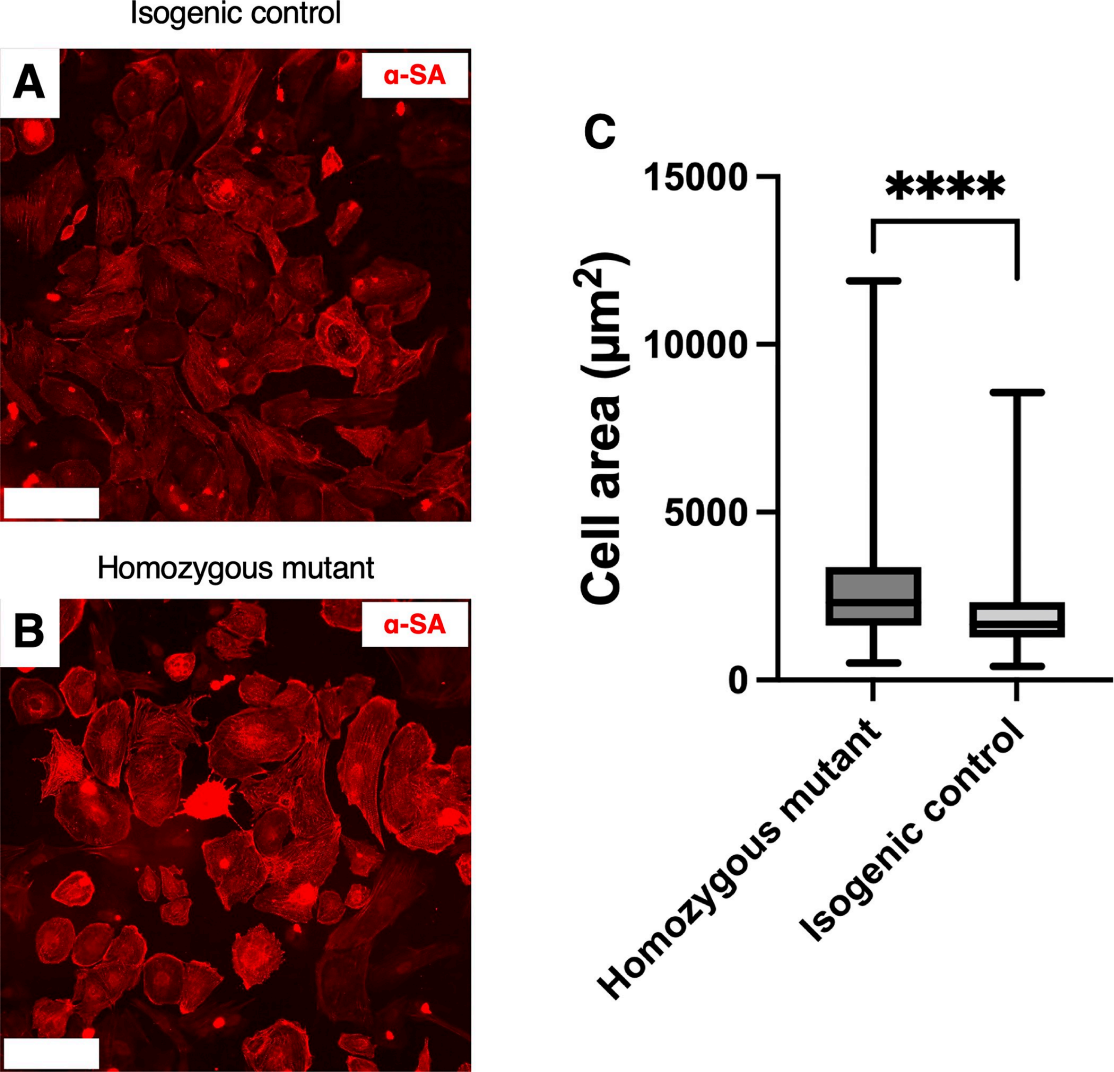
Cell size of induced pluripotent stem cell-derived cardiomyocytes from homozygous mutant and isogenic control human iPSCs. Representative alpha-sarcomeric actin-stained isogenic control and homozygous mutant cells used for cell size analysis (α-SA, red fluorescence, A and B respectively). The area of homozygous mutant iPSC-CMs was approximately 40% more than isogenic control iPSC-CMs (C, n = 3 homozygous mutant lines, total 268 cells, and 3 isogenic control lines, total 301 cells, p < 0.001). Whiskers indicate the maximum and minimum values; boxes show the mean and inter-quartile range.

Next, we examined the size (surface area) of the iPSC-CMs derived from homozygous mutant and isogenic control iPSCs using immunocytochemistry. The area of iPSC-CMs derived from homozygous mutant iPSCs was approximately 40% larger than the isogenic control iPSC-CMs (2733 ± 99.13 μm^2^ versus 1957 ± 63.13 μm^2^, p < 0.001, Fig 4).

### Homozygous mutant iPSC-CMs have impaired relaxation and are hypocontractile

**Fig 5:**
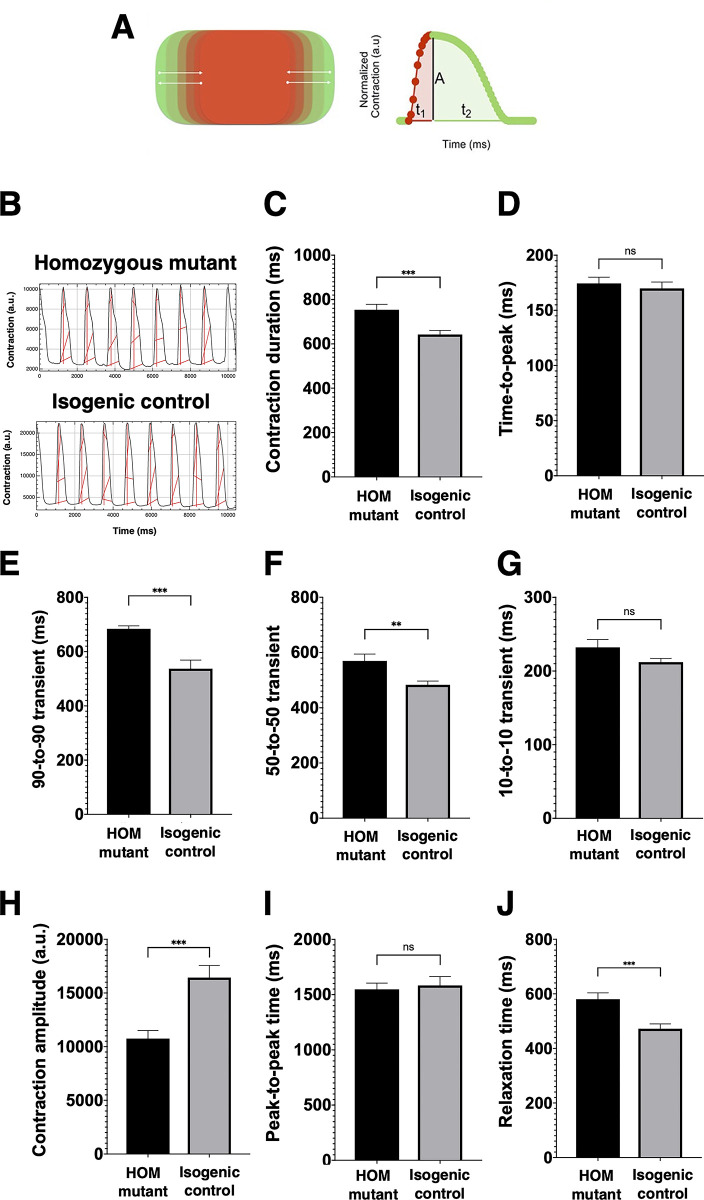
Contraction kinetics of induced pluripotent stem cell-derived cardiomyocytes (iPSC-CMs) with and without the R820W mutation analysed by MUSCLEMOTION software. Panel A shows a schematic of the contractile pattern of an artificial cell the develops used to test the MUSCLEMOTION and relative parameters corresponding to amplitude of contraction (A), time-to-peak (t_1_), and relaxation time (t_2_). This figure was adapted and reproduced here from the report by Sala *et al*. with the authors permission [26]. Panel B shows contraction of iPSC-CMs as graphical representation of pixel motion (n = 3 homozygous mutant lines and 3 isogenic control lines). The red lines indicate measure of contraction amplitude and duration. Contraction duration (C), time-to-peak (D), 90-to-90 transient (E), 50-to-50 transient (F), 10-to-10 transient (G), contraction amplitude (H), peak-to-peak time (I) and relaxation time (J) are shown. HOM = homozygous. Error bars represent the mean ± SEM. Significance was determined by Student’s T-test; *** = p < 0.0001, ** = p < 0.001, ns = not significant. a.u. = arbitrary units.

Since alterations in contractility and relaxation kinetics are commonly reported consequences of HCM-causing mutations, we analysed these parameters of our iPSC-CMs using the image tracking software MUSCLEMOTION (Fig 5A and 5B). Compared to isogenic control iPSC-CMs, homozygous mutant iPSC-CMs had a longer contraction duration (753.9 ± 24.72 ms versus 642.4 ± 18.43 ms, p < 0.001, Fig 5C), 90–90 transient (684.2 ± 10.89 ms versus 537.2 ± 31.56 ms, p < 0.001, Fig 5E), 50-to- 50 transient (569.7 ± 24.59 ms versus 483.0 ± 13.78 ms, p = 0.0031, Fig 5F), relaxation time (580.2 ± 23.46 ms versus 472.1 ± 17.84 ms, p < 0.001, Fig 5J) and a reduced contraction amplitude (10749 ± 758.2 a.u. versus 16434 ± 1139 a.u., p < 0.001, Fig 5H). Overall, these results indicate that homozygous mutant iPSC- CMs have impaired relaxation and are hypocontractile.

## Discussion

In this study we first introduced the Ragdoll *MYBPC3*/R820W mutation into a human iPSC line, differentiated to cardiomyocytes and used a number of assays to compare homozygous mutant iPSC-CMs to isogenic control iPSC-CMs. The aim was to create a HCM disease model that could act as a resource to facilitate future investigations into the molecular effects of the *MYBPC3*/R820W mutation and ultimately develop therapeutic drug screening. Using qPCR, ELISA and immunocytochemistry we show that homozygous mutant iPSC-CMs produce *MYBPC3* mRNA in levels similar to isogenic control iPSC-CMs, with similar cMyBP-C protein levels and that cMyBP-C is incorporated into the A-band of the sarcomere as expected [30]. In addition, we demonstrate increased cell size of homozygous mutant iPSC-CMs compared to isogenic control cells and show that homozygous mutant cells display impaired relaxation kinetics and hypocontractility, therefore confirming this homozygous mutant cell line as a potentially valuable feline HCM disease model. In human HCM research there have been numerous studies investigating the molecular effects of mutations in HCM using an iPSC-CM model and CRISPR/Cas9 gene editing technology to help exclude the confounding variable of genetic background. The authors acknowledge that this work does not further elucidate specific molecular mechanisms driving HCM in cats, however the same cellular models do not currently exist for feline mutations. Therefore, the work presented in this study acts as an initial step towards further investigations into the molecular mechanisms driving HCM in feline patients.

When the expression of *MYBPC3* in homozygous mutant iPSC-CMs was examined, there was comparable RNA and protein levels to the isogenic control cells. Additionally, mutant protein was incorporated into the sarcomere and did not appear to disrupt the highly organised sarcomere structure. This is important because most of the >200 identified mutations in *MYBPC3* that cause HCM encode for truncated proteins, with transcripts targeted for nonsense-mediated decay (NMD) and mutant protein cleared through UPS, causing haploinsufficiency [11]. In cases where *MYBPC3* missense mutations do not code for a premature termination codon (PTC), misfolded proteins can be targeted for degradation causing reduced protein incorporated into the sarcomere and disruption to sarcomere function. For example, the human HCM-linked W792R mutation in *MYBPC3* reduces the thermal stability of domain C6, causing the region to unfold and expose a proteolytic cleavage site, with degradation of the cleaved protein completed through UPS [31]. This also leads to a reduction in cMyBP-C incorporated into sarcomeres, which in the study by Smelter *et al*. was identifiable by immunocytochemistry. Our homozygous mutant and isogenic control iPSC-CMs had comparable immunostaining intensities for cMyBP-C and similar amounts of cMyBP-C when measured by ELISA, making it unlikely that there is significant degradation of the protein associated with the *MYPBC3*/R820W mutation. We therefore propose that the pathogenesis of this mutation is associated with altered function of the protein rather than haploinsufficiency, as previously described in a homozygous Ragdoll cat [5].

In support of this proposition, a separate human HCM-linked mutation in codon 820 of *MYBPC3*, R820Q (a arginine to glutamine substitution) was found to not disrupt protein folding but instead affect protein-protein interactions [16]. This pathogenesis may also explain why cats that are heterozygous for R820W exhibit a mild phenotype compared to homozygous cats, since normal cMyBP-C produced from the healthy allele would ameliorate damaging effects of the mutant allele products [5]. De Lange *et al*. investigated another missense mutation in *MYBPC3*, E258K (a glutamate to lysine substitution in codon 258). They found that the mutant protein could be incorporated into the sarcomere but that the mutation disrupted the interaction between cMyBP-C and myosin, altering contraction kinetics and compromising the twitch force of engineered cardiac tissue [32]. These studies, along with our findings, suggest that R820W and other missense mutations in *MYBPC3* cause HCM through altering protein function rather than causing haploinsufficiency. This could be assessed in future studies by conducting further experiments analysing the cell contractile function, for example by measuring force of contraction or Ca^2+^ handling by live cell immunofluorescence under field stimulation conditions [33, 34].

Our results of cell area show that the R820W mutation causes cellular hypertrophy. Although we did not investigate possible mechanisms leading from mutation to increased cellular size, hypertrophy is commonly documented in HCM causing mutations when studied at the single cell level [7, 24, 35]. Several signalling pathways have been implicated in the development of cardiomyocyte hypertrophy. Cohn *et al*., using an iPSC-CM model to investigate the molecular effects of a HCM-causing *MYH7* mutation, found increased levels of phosphorylated ERK2 and AKT [24]. Phosphorylated ERK2 and AKT have numerous cytoplasmic and nuclear targets including activation of transcription factors involved in cell growth, proliferation and hypertrophy [36, 37]. Another study using an iPSC-CM model of HCM found the transcriptional activator pathway calcineurin-NFAT played a role in the development of cellular enlargement, via similar targets as ERK2 and AKT such as activation of GATA4 [35]. GATA4 is a key regulator of cardiac specific gene expression and had been shown to be critical to the hearts ability to undergo hypertrophy in response to physiological stimuli [38]. Further characterisation of how the R820W mutation specifically causes cellular hypertrophy would therefore be warranted and may elucidate novel therapeutic targets.

Analysis of the contractile properties of homozygous mutant and isogenic control iPSC-CMs found that the R820W mutation caused hypocontractility and impaired relaxation. Impaired relaxation is a common phenotypic finding among HCM-causing mutations and manifests at the organ level as diastolic dysfunction, one of the hallmark clinical features of HCM in both cats and humans [6, 24, 39, 40]. Although we did not investigate the calcium handling characteristics of our cells, previous studies suggest that altered calcium homeostasis and sensitivity of the myofilament to calcium contributes to impaired relaxation. Wu *et al*. used an iPSC-CM model of HCM to show that four different HCM-linked mutations in three sarcomeric genes (*MYH7*, *MYBPC3* and *TNNT2*) cause diastolic dysfunction by increasing both diastolic calcium concentrations and sensitivity of the myofilament to calcium [41]. Both hypocontractility and hypercontractility of cardiomyocytes have been reported in iPSC-CM HCM models. Multiple studies on different HCM causing mutations find that the result of increased myofilament calcium sensitivity is enhanced contractility [24, 34, 42–46]. On the other hand, Mosqueira *et al*. found that iPSC-CMs harbouring the HCM- causing R453C mutation in *MYH7* had a reduced contraction force and this was accompanied by higher metabolic respiration activity [7]. This finding is consistent with an energy depletion model of HCM, where sarcomeric mutations cause inefficient ATP usage, causing increases in the cell energy demand, and reducing energy available to bring the calcium concentration in the cytosol back to baseline levels. This precipitates arrhythmogenesis and causes impaired relaxation. Further investigation is warranted into the effects of the R820W mutation on contractility, for example by using engineered heart tissue (EHTs) and subjecting cardiomyocytes to loading to simulate conditions within the heart.

There are some limitations associated with this study. First, we used a human iPSC line as although we have also developed a feline iPSC line, this requires further optimisation prior to gene editing and differentiation [47]. Whilst human and feline HCM are almost analogous diseases, there remains the possibility that there are species differences that may hamper the applicability of this human system to the feline species. The pluripotency of the iPSCs after gene editing was confirmed, however further analysis could be performed to confirm a normal karyotype and assess the cardiac differentiation efficiency of both isogenic control and homozygous mutant cells. Additionally, whilst we carefully designed guide RNA to minimise off-target effects, which was confirmed by off-target prediction models, whole genome sequencing of each line should be performed prior to undertaking investigations into disease mechanism. In addition, iPSC-CMs were not under field stimulation (usually at 1 Hz) when videos were taken for analysis by MUSCLEMOTION software. This is not ideal since variable beat rates between iPSC-CM preparations can alter parameters associated with contraction kinetics, although the peak-to-peak time (a measure of beating rate) was not different between the isogenic control and homozygous mutant iPSC-CMs. Further work including the use of calcium-based imaging would provide further understanding of how the R820W mutation alters the function of cardiomyocytes. In addition, we anticipate future work utilising this cell line harbouring the feline HCM causing R820W mutation will further expand on molecular mechanisms of this disease by using techniques such as RNA sequencing, proteomics, mitochondrial function, and oxidative stress assays. Specifically, since a wide variety of pathways have been implicated in the genotype to phenotype relationship, a multiscale approach first starting with transcriptomic data to assess differentially expressed pathways, and then moving on to investigating these pathways at the protein, myofibril, cell, and tissue level, including engineered heart tissues, would be required to elucidate mechanistic pathways, which was beyond the scope of the current work.

## Conclusions

One of the major difficulties in studying HCM pathogenesis is disease heterogeneity and impact of genetic background on phenotype expression. We overcame this hurdle by using CRISPR/Cas9 gene edited human iPSCs to create isogenic cell lines containing the missense R820W mutation in MYBPC3. Our work indicates that this mutation, which causes a severe HCM phenotype when expressed in homozygosity in Ragdoll cats and humans, causes altered contraction kinetics and cellular hypertrophy in this cellular model. We anticipate that this disease model of HCM will provide a valuable resource to gain further insight into the disease-causing molecular events and ultimately identify novel therapeutic targets.

## Supporting information

S1 Fig Pluripotent markers and trilineage differentiation of induced pluripotent stem cells. Prior to gene editing, the iPSCs showed expression of pluripotent markers *NANOG*, SSEA4, *SOX2* and Tra-1-60 (panel A). Following gene editing, the iPSC retain expression of these markers (panel B). Additionally, iPSC post-editing spontaneously differentiated into cells representative of the three germ layers after embryoid-body formation, namely mesoderm (smooth muscle actin, green fluorescence), ectoderm (βIII-tubulin, red fluorescence) and endoderm (insulin, green fluorescence), panel C. Representative images from three isogenic controls and three homozygous mutant lines. Scale bar = 200μm except panel C, insulin stain = 100μm.
(TIF)
