# Supplementary figures and images for: CRISPR/Cas9 gene editing in induced pluripotent stem cells to investigate the feline hypertrophic cardiomyopathy causing MYBPC3/R820W mutation
Source: PLoS One. 2024 Oct 10;19(10):e0311761. doi: 10.1371/journal.pone.0311761 (PMC11466433; doi:10.1371/journal.pone.0311761)

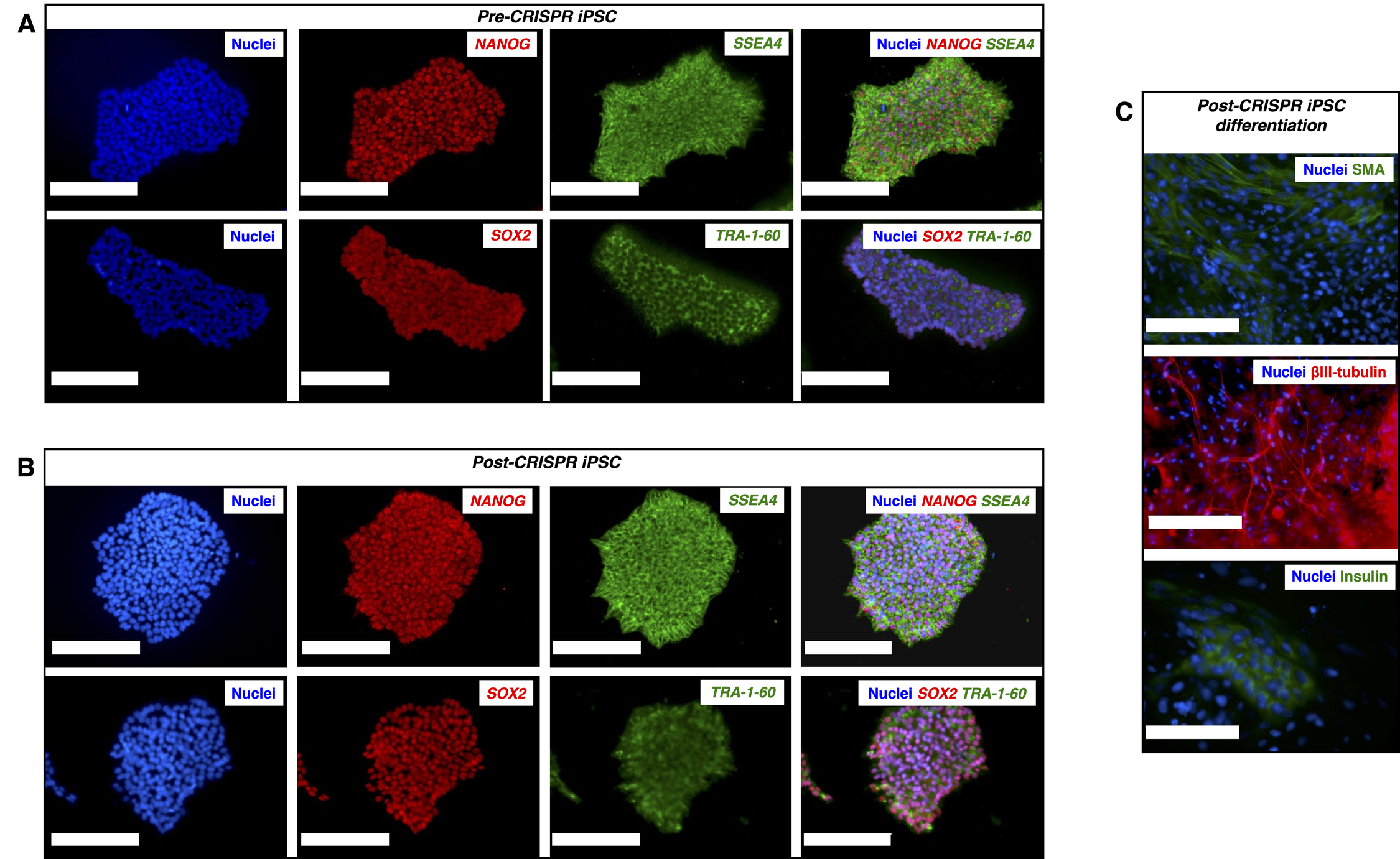

Supplement: S1 Fig — Prior to gene editing, the iPSCs showed expression of pluripotent markers NANOG, SSEA4, SOX2 and Tra-1-60 (panel A). Following gene editing, the iPSC retain expression of these markers (panel B). Additionally, iPSC post-editing spontaneously differentiated into cells representative of the three germ layers after embryoid-body formation, namely mesoderm (smooth muscle actin, green fluorescence), ectoderm (βIII-tubulin, red fluorescence) and endoderm (insulin, green fluorescence), panel C. Representative images from three isogenic controls and three homozygous mutant lines. Scale bar = 200μm except panel C, insulin stain = 100μm. (TIF) [file pone.0311761.s001.tif]
